# Evaluating the Potential for Resistance Development to Antimicrobial Blue Light (at 405 nm) in Gram-Negative Bacteria: *In vitro* and *in vivo* Studies

**DOI:** 10.3389/fmicb.2018.02403

**Published:** 2018-10-16

**Authors:** Leon G. Leanse, Olivia D. Harrington, Yanyan Fang, Imran Ahmed, Xueping Sharon Goh, Tianhong Dai

**Affiliations:** Wellman Center for Photomedicine, Massachusetts General Hospital, Harvard Medical School, Boston, MA, United States

**Keywords:** antimicrobial blue light, serial exposure, antimicrobial resistance, Gram-negative bacteria, wound infection

## Abstract

Antimicrobial resistance is a threat to public health that requires our immediate attention. With increasing numbers of microbes that are becoming resistant to routinely used antimicrobials, it is vital that we look to other, non-traditional therapies for the treatment of infections. Antimicrobial blue light (aBL) is an innovative approach that has demonstrated efficacy for the inactivation of an array of microbial pathogens. In the present study, we investigated the potential for resistance development to aBL in Gram-negative pathogenic bacteria by carrying out multiple aBL exposures on bacteria. In the first aBL exposure, clinical isolates of *Pseudomonas aeruginosa*, *Acinetobacter baumannii*, and uropathogenic *Escherichia coli* [10^7^ colony forming units/mL (CFU/mL)] were irradiated in phosphate-buffered saline with aBL at 405 nm until a >99.99% reduction in bacterial viability was achieved. Irradiation was then repeated for each bacterial species over 20 cycles of aBL exposure. The potential for resistance development to aBL was also investigated *in vivo*, in superficial mouse wounds infected with a bioluminescent strain of *P. aeruginosa* (PAO1; 10^8^ CFU) and irradiated with a sub-curative radiant exposures of 108 or 216 J/cm^2^ aBL over 5 cycles of treatment (over 5 days) prior to bacterial isolation from the animal tissue. PAO1 isolated from infected tissue were treated with aBL at 216 J/cm^2^, *in vitro*, in parallel with unexposed PAO1 or PAO1 isolates from mouse wound infections not treated with aBL. No statistically significant correlation was found between the aBL-susceptibility of bacteria *in vitro* and the number of cycles of aBL exposure any bacterial species (*P* ≥ 0.26). In addition, serial exposure of infected mouse wounds to aBL did not result in any change in the susceptibility to aBL of PAO1 (*P* = 0.97). In conclusion, it is unlikely that sequential exposure to aBL will result in aBL-resistance in Gram-negative bacteria. Also, multiple aBL treatments may potentially be administered to an infected wound without resistance development becoming a concern.

## Introduction

Antimicrobial resistance (AMR) is a threat to public health that requires our immediate attention. The development of resistance to antimicrobials is, in part, a result of overexposure, which, in turn, leads to the positive selection of mutations that incur a resistance phenotype (Kohanski et al., 2010). Examples of mechanisms of resistance include expression of enzymes that break down antimicrobials, e.g., beta-lactamases, against beta-lactam antibiotics, or the expression of efflux pumps, e.g., TetA efflux pump, against tetracycline (Bush and Jacoby, 2010; Møller et al., 2016). Consequently, increasing numbers of microbes are becoming resistant to routinely used antimicrobials, with some developing resistance to all forms of traditional therapy (McCarthy, 2017). Therefore, it is vital that we look to other “non-traditional” approaches to reduce any ill-effects that may result from AMR.

Antimicrobial blue light (aBL; at 405 nm) is a novel strategy that has demonstrated efficacy against a variety of microbial pathogens both *in vitro* and *in vivo*, regardless of their AMR status (Dai et al., 2012; Zhang et al., 2014; Hessling et al., 2017; Tomb et al., 2017; Wang et al., 2017). However, due to the limited studies that have investigated the potential for resistance development to aBL by microbial pathogens, it is difficult to predict whether future aBL-resistance development will become a concern. Presently, the studies that have evaluated the potential for resistance development to aBL have suggested it is unlikely to occur through serial exposure (Zhang et al., 2014; Amin et al., 2016; Tomb et al., 2017). The only investigation that has suggested the potential for resistance development to aBL was a study carried out by Guffey et al. (2013), which found that *Staphylococcus aureus* developed resistance following 7 cycles of aBL exposure (Guffey et al., 2013). Therefore, whether bacteria may develop resistance to aBL through serial exposure remains a question. While resistance development to aBL in bacteria has been investigated *in vitro* by several groups, there has never been an evaluation regarding the potential for resistance development resulting from multiple aBL treatments of bacterial infections *in vivo*. This is paramount to clinically predict whether multiple aBL treatments will increase the chance of inducing aBL resistance, which in turn can inform treatment strategies.

In the present study, we investigated the effect of 20 cycles of aBL exposure (resulting in a >99.99% CFU reduction) on bacteria, *in vitro*, on aBL activity against three clinically important Gram-negative pathogenic bacteria; including multidrug-resistant clinical isolates of *Pseudomonas aeruginosa*, *Acinetobacter baumannii*, and uropathogenic *Escherichia coli*. Additionally, for the first time, we investigated the potential for resistance development to aBL from multiple cycles of aBL exposure to mouse skin abrasion wounds infected with a bioluminescent variant of the model organism *P. aeruginosa*.

## Materials and Methods

### Blue Light Source

**FIGURE 1:**
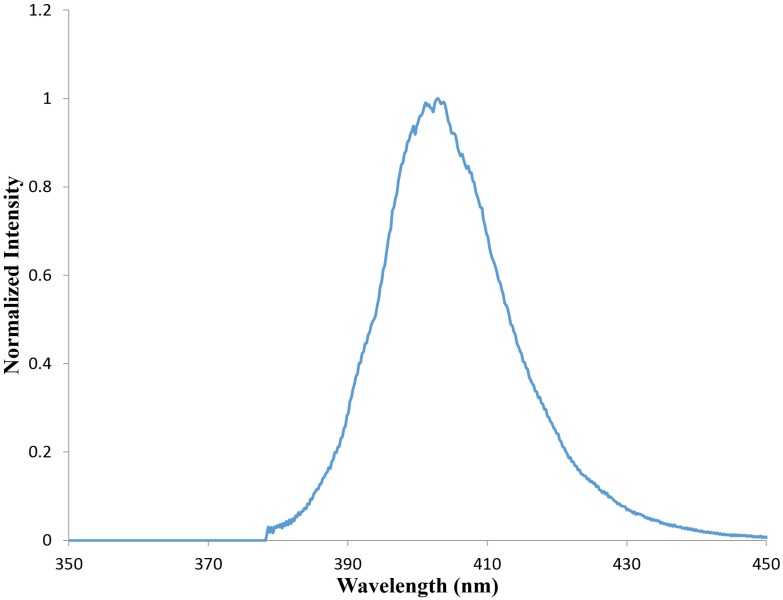
Emission spectrum from M405L3 LED (Data taken from Thorlabs.com) showing a peak emission at 405 nm.

aBL was irradiated with a light emitting diode (LED; M405L2, Thorlabs, Newton, NJ, United States) with a peak emission of 405 nm (**Figure 1**). The irradiance was controlled by adjusting the distance of the aperture of the LED and the target with the use of a power/energy meter (PM100D; Thorlabs, Newton, NJ, United States).

### Bacterial Strains

The bacterial strains used in this study included multidrug-resistant clinical wound isolates of *A. baumannii* and *P. aeruginosa* (strain AF 0005 and AF 0001, respectively – kind gifts from Dr. Clinton Murray at Brooke Army Medical Center, Fort Sam Houston, TX, United States), and a uropathogenic strain of *E. coli* (UTI 89 – a kind gift from Dr. Patrick C. Seed at the Duke University School of Medicine) (Balsara et al., 2013). In addition, a bioluminescent variant of *P. aeruginosa* containing a chromosomally integrated *lux* operon from *Photorhabdus luminescens* (strain PAO1, a kind gift from Dr. Joanna B. Goldberg at the Emory University School of Medicine) (Damron et al., 2013) was screened for resistance development to aBL during *in vivo* studies; allowing real-time monitoring of bioluminescence from bacteria. Bacteria were cultured in brain heart infusion (BHI) agar plates and incubated at 37°C or in BHI broth in an orbital incubator (37°C; 180 rpm).

### Dose-Response Analysis on Planktonic Bacteria *in vitro*

To determine the appropriate radiant exposure of aBL for *in vitro* serial aBL exposure, bacteria were initially irradiated with different doses of aBL. In brief, a single colony of bacteria was cultured overnight in 3 mL BHI broth and then centrifuged at 3,500 ×*g* for 10 min just prior to aBL exposure. The bacterial pellets were then re-suspended in 3 mL phosphate buffered saline (PBS; 0.01 M phosphate buffer, 0.0027 M KCL, and 0.137 M NaCl; pH 7.4), adjusted to approximately 10^7^ colony forming units (CFU)/mL, and subsequently transferred to a 35 × 12 mm petri dish. aBL was then delivered at an irradiance of 0.1–0.15 W/cm^2^ to the bacterial suspension for up to 96 min [Radiant exposure = irradiance (W/cm^2^) × time (s)]. During aBL irradiation, the bacterial suspension was stirred using a 12-mm magnetic bar (20 rpm) to ensure uniform exposure of cells. Aliquots (20 μL) of the suspension were withdrawn post-exposure to aBL at varying time points and the CFUs were determined by serial dilution on BHI agar plates as described previously (Jett et al., 1997). Experiments were performed in triplicate (biological replicates).

### Serial aBL Exposures on Bacteria *in vitro*

Following a 99.99% reduction in bacterial CFU (4 log_10_ CFU), a single colony from the surviving bacteria, was subsequently inoculated into 3 mL BHI overnight and then exposed to aBL again in the same manner as described above. This was performed until 20 cycles of exposure were reached. For each bacterial strain, the radiant exposure of aBL was kept constant throughout of the 20 cycles of passage. All experiments were performed in triplicate (biological replicates).

### Mouse Model of Skin Abrasion Infection

Female BALB/c mice aged 6–8 weeks and weighing 17–19 g were purchased from Charles River Laboratories (Wilmington, MA, United States). All animal procedures were approved by the Institutional Animal Care and Use Committees of Massachusetts General Hospital (protocol no: 2015N000187) in accordance with National Institute of Health guidelines. Prior to producing the skin abrasion wounds, mice were anesthetized through intraperitoneal injection of a ketamine/xylazine cocktail (20–100 mg/kg). The mice were then shaved, and the tissue was abraded within a defined 1.0-cm × 1.0-cm area using a #15 sterile scalpel blade (Dai et al., 2013). The scraped area did not yield any blood. Within 5 min of producing the abrasions, approximately 10^8^ CFU of bacteria in PBS (60 μL) were inoculated into each wound. A bioluminescent variant of *P. aeruginosa* (strain PAO1) was used as the model organism for this study.

### Serial aBL Exposures on Bacteria in Mouse Skin Abrasion Wounds

To determine whether multiple aBL treatments of infected mouse abrasion wounds would result in a resistance development to aBL, infected mouse wounds were first subjected to 5 cycles of aBL irradiation (108 or 216 J/cm^2^). In brief, 3 h after inoculating the bacterial suspension, aBL was irradiated at 0.1 W/cm^2^, until a total radiant exposure of 108 or 216 J/cm^2^ was delivered, ensuring a still active infection post-treatment (indicated by bioluminescence imaging – see below; **Figure 4A**). Mouse skin abrasion infections were exposed to aBL over 5 cycles of treatment spanning a total of 5 days. Untreated controls were also included. Bioluminescence imaging was carried out after each aliquot of aBL to ensure the presence of actively replicating bacteria. For each condition including untreated controls, a group of five mice were used.

### Isolation of Bacteria From Infected Mouse Tissue

Following 5 cycles of aBL exposure, the bacteria were isolated from the aBL-treated infected tissue and subjected to aBL irradiation *in vitro*, to ascertain whether any aBL resistance developed. To carry this out, the skin tissue was first excised from the 1.0-cm × 1.0-cm infected area from the mice exposed to sub-curative aBL radiant exposures of 108 or 216 J/cm^2^ and untreated mice using a #15 sterile scalpel blade. Sterile PBS (1 mL) was added to the excised skin which was then homogenized using a #15 sterile scalpel by gently cutting up the tissue. The PBS solution containing the isolated bacterial suspension was subsequently transferred to a 2 mL microcentrifuge tube (Eppendorf) and bioluminescence imaging was carried out to ensure adequate isolation of PAO1 into the PBS. To evaluate aBL-susceptibility of the PAO1 isolated from wound infections from each mouse, the bacteria were then subjected to aBL treatment *in vitro* with a radiant exposure of 216 J/cm^2^, as described above. The PAO1 wound isolates were then inoculated onto BHI agar following serial dilution of the PBS inoculum for CFU enumeration.

### Bioluminescence Imaging

The bioluminescence of bacteria in mouse skin abrasion wounds was detected by using an IVIS Lumina II In Vivo Imaging System (PerkinElmer, Inc., Hopkinton, MA, United States). This system has an adaptable field of view ranging from 5–12.5 cm and a 24-cm lens for imaging of up to five mice simultaneously. The IVIS Lumina II has a CCD camera that is 13 × 13 mm square, with 1024 × 1024 pixels 13 microns in width, to permit high resolution when imaging. The system was operated using the Living Image software, for image collection and photon counting for relative luminescence unit (RLU) quantification in real time.

### Statistical Analyses

Where necessary, correlation coefficients were calculated (Pearson r) where −1 or +1 indicated a strong negative or positive relationship, respectively, and 0 indicated no relationship; *P* < 0.05 was considered significant. When necessary, an unpaired *t*-test or one-way analysis of variance (ANOVA) was carried out where a *P* < 0.05 was considered significant. All statistical analyses were performed on GraphPad prism.

## Results

### Varying Susceptibility to aBL Inactivation Was Elicited by Different Bacterial Species

**FIGURE 2:**
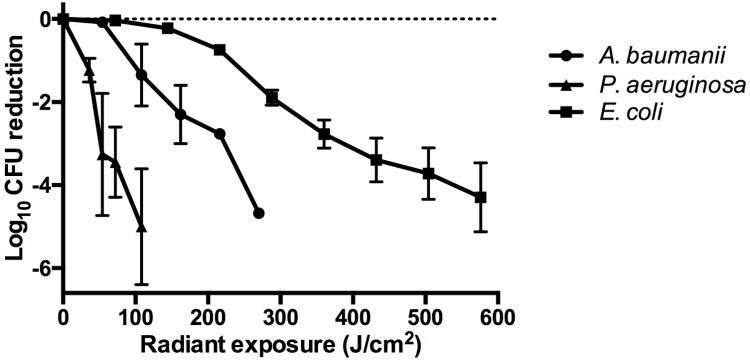
Dose-response curves illustrating the log_10_ CFU reduction for *A. baumannii*, *P. aeruginosa*, and *E. coli* following exposure to increasing doses of aBL. Error bars: standard deviation between replicates.

The dose-response analyses from exposure of bacteria to aBL revealed varying sensitivities elicited by each strain (**Figure 2**). The more sensitive strains were *P. aeruginosa* and *A. baumannii*, which resulted in a 5-log_10_ and 4.69-log_10_ CFU reduction, respectively, following 144 and 270 J/cm^2^ exposure, respectively. At similar radiant exposures (144 J/cm^2^), only a 0.22-log_10_ CFU reduction was observed in the *E. coli* strain. For *E. coli*, to achieve a higher CFU reduction as compared to *A. baumannii* or *P. aeruginosa*, a radiant exposure of 576 J/cm^2^ was required, which inactivated 4.29-log_10_ CFU.

### Development of aBL-Resistance by Bacteria *in vitro* Did Not Occur Following 20 Cycles of aBL Inactivation

**FIGURE 3:**
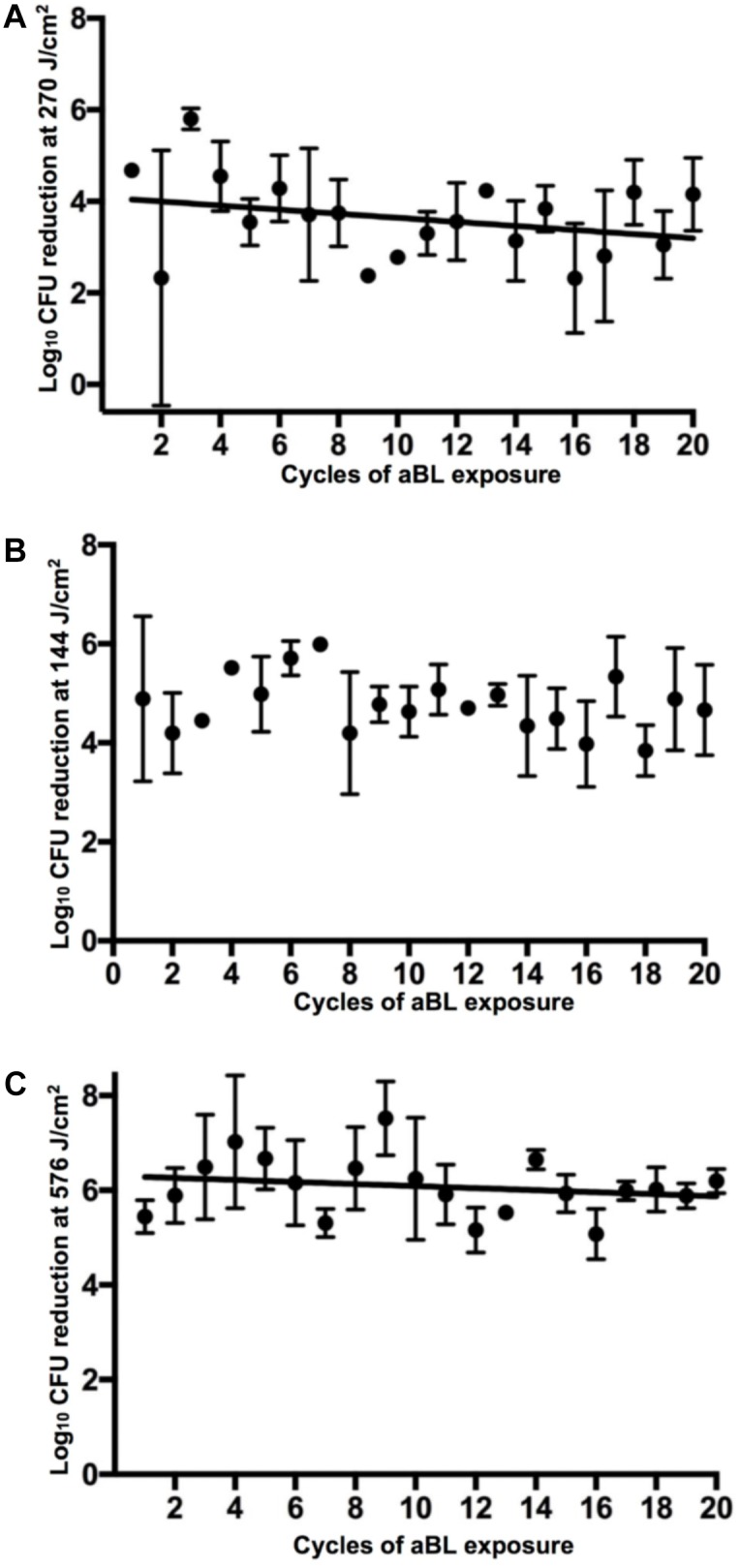
log_10_ CFU reduction of bacteria in 20 successive cycles of aBL inactivation. **(A)** *A. baumannii*, **(B)** *P. aeruginosa*, and **(C)** *E. coli*. Error bars: standard deviation.

Exposure to aBL over 20 cycles of treatment did not appear to affect the susceptibility to aBL in any bacterial species (**Figure 3**). Correlation analyses revealed correlation coefficients of −0.30, −0.17, and −0.21 (*P* ≥ 0.26), in *A. baumannii*, *P. aeruginosa*, and *E. coli*, respectively. Thus demonstrating that in every species tested, there was no statistically significant relationship between the numbers of aBL exposures and log_10_ CFU reduction and resistance development by bacteria did not occur. An unexpected finding was the variability of log_10_ CFU reduction occurring within cycles of exposure, where certain cycles were significantly more resistant than others. For example, a statistically significant increase in aBL-tolerance is evident when Cycle 1 is compared with Cycle 9, 16, and 17 in *A. baumannii* (*P* < 0.001). However, this comparative “resistance” phenotype appears unstable as upon cycle, bacteria revert to being sensitive post-cycle.

### Development of aBL-Resistance by Bacteria in Infected Mouse Skin Abrasions Did Not Occur After 5 Cycles of Exposure to aBL

**FIGURE 4:**
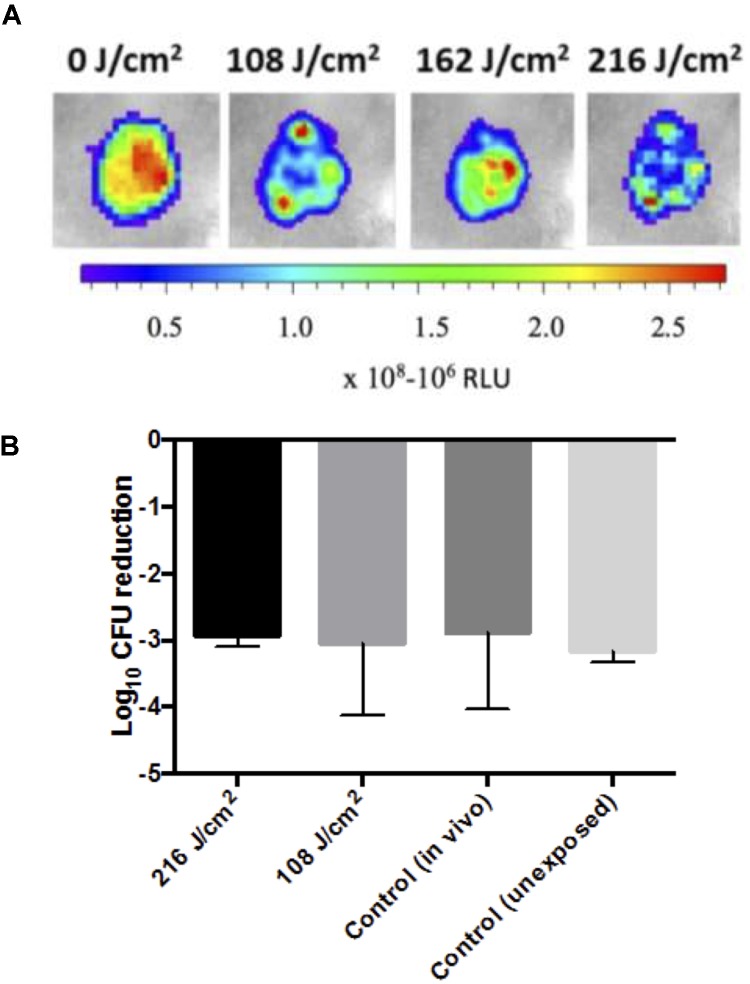
**(A)** Representative photos of PAO1 infected sections following sub-curative aBL treatment (108–216 J/cm^2^) with a color bar indicating relative luminescence units (RLU). **(B)** Bar-graphs showing log_10_ CFU reduction in response to 216 J/cm^2^ aBL of PAO1 isolated from mouse infected tissue that was either untreated [control (*in vivo*)] or previously exposed to 108 or 216 J/cm^2^ over 5 cycles of treatment, and a control PAO1 that was not previously exposed to aBL. Error bars: standard error of the mean.

PAO1 infected mouse abrasion wounds were subjected to 5 cycles of aBL treatment at radiant exposures of 108 or 216 J/cm^2^ (**Figure 4A**). Post-exposure to aBL, the infecting PAO1 strain was isolated from the tissue and subsequently treated *in vitro* with 216 J/cm^2^ aBL. Following *in vitro* exposure, the isolated PAO1 strain did not demonstrate any marked increase in resistance to aBL, when compared to non-aBL exposed and non-treated *in vivo* controls. This is evidenced by the absence of statistically significant changes to the log_10_ CFU reduction following the *in vitro* 216 J/cm^2^ aBL exposure, compared with unexposed PAO1 or PAO1 isolated from infected mouse skin not treated with aBL (**Figure 4B**; *P* = 0.97).

## Discussion

Resistance development to aBL has been investigated previously by our group, where we have not found a correlation between serial aBL exposure and resistance development in multiple bacterial pathogens (Zhang et al., 2014; Amin et al., 2016). These investigations involved exposing the bacteria to aBL over 10 cycles. A study by Tomb et al. (2017) investigated the potential for resistance development in the Gram-positive species, *S. aureus*, which involved serial exposure to aBL over 15 cycles, where they did not find a correlation between serial aBL exposure and resistance development (Tomb et al., 2017). In the present study, we investigated the potential for resistance development to aBL in 3 separate Gram-negative bacterial species including multidrug-resistant clinical isolates over 20 cycles of aBL exposure, *in vitro*. In addition, for the first time, the potential for resistance development to aBL was investigated *in vivo*, through serial exposure of *P. aeruginosa* infected mouse skin abrasion wounds to aBL over 5 cycles of treatment.

Our findings illustrate that different bacterial species elicit variable sensitivities to aBL (**Figure 2**) with *P. aeruginosa* and *A. baumannii* being sensitive to aBL with 5-log_10_ and 4.69-log_10_ CFU being inactivated following 144 and 270 J/cm^2^ treatment, respectively. *E. coli*, however, is comparatively more resistant, with 576 J/cm^2^ being required to inactivate 4.29-log_10_ CFU. These findings are comparable to previous data reported by Gupta et al. (2015).

Serial aBL exposures over 20 cycles *in vitro* did not reveal a correlation between aBL serial exposure and resistance development (**Figure 3**). However, as the study was limited to serial exposure of a single colony (per biological replicate), this may not be representative of the entire bacterial population. Our findings, however, are supported by previous studies that have investigated resistance development *in vitro* over fewer cycles (Zhang et al., 2014; Amin et al., 2016; Tomb et al., 2017). In contrast, a study by Guffey et al. (2013) found that following 7 cycles of exposure of *S. aureus* to aBL *in vitro*, resistance to aBL did develop; although, due to the fewer cycles of passage, these findings are not entirely comparable and therefore we cannot conclude that this resistance phenotype was stable.

An interesting finding of our study, however, was that at certain cycles of exposure statistically significant sporadic higher tolerances of bacteria to aBL were observed, which appeared transient. This might explain the findings from Guffey et al. (2013) where resistance development was apparent at the 7th cycle of treatment. A possible explanation for this “transient-resistance” phenotype were changes to the bacterial transcriptome following multiple cycles of aBL irradiation. aBL exposure over multiple cycles may have resulted in the stress induced overexpression of superoxide dismutases (e.g., SodC) that inactivate generated ROS and/or genes involved in opposing the any damage that may have resulted from ROS generation (e.g., *oxyR* or SOS regulons) (Asad et al., 1997; Wang et al., 2018). In addition, a recent study carried out by Fyrestam (2017) illustrated that the serial passages of the periodontal bacterial pathogens, *Aggregatibacter actinomycetemcomitans* and *Porphyromonas gingivalis* resulted in changes to the relative abundances of endogenous porphyrins (Fyrestam, 2017). Therefore, the transient changes in the aBL-sensitivities, may have arisen because of differential concentrations of endogenous porphyrins resulting from serial passage during aBL exposure. Further work, investigating antioxidant expression (e.g., *oxyR*) resulting from serial aBL exposure or quantification of the relative concentrations of endogenous porphyrins within bacteria at different cycles of exposure, would be necessary to corroborate these hypotheses.

Following 5 cycles of aBL exposure to infected mouse wounds (**Figure 4A**) no effect on the susceptibility of the PAO1 strain to aBL was observed, suggesting that serial exposure of aBL to an infected wound is unlikely to result in resistance (**Figure 4B**). However, as the study was limited to 5 cycles of sequential treatment, there remains the possibility that increasing the number of aBL exposures may induce resistance development. In addition, the lack of resistance development during the 5 cycles treatment of an active infection where the bacteria are replicating, suggests that growth in the presence of aBL is unlikely to result resistance development. However, further work investigating the effect of continuous exposure on resistance development, is required for confirmation. The lack of resistance development to aBL following multiple cycles of treatment, suggests that the genes that are involved in mediating aBL activity are additionally important for fundamental processes occurring in the cell. Many of the porphyrins produced in bacteria are formed during heme biosynthesis which is essential for bacterial survival and pathogenesis (Choby and Skaar, 2016) and thus unlikely to incur mutation; as per the knock out rate hypothesis that stipulates that the dispensability of a gene is correlated with loss of function (Hurst and Smith, 1999).

## Conclusion

In conclusion, it does not appear likely that aBL-resistance can be induced in Gram-negative bacteria through multiple cycles of aBL exposure *in vitro* or *in vivo*. However, it is important to consider that the *in vitro* serial exposure was only limited to a representative bacterial population, and thus we cannot categorically conclude that resistance development cannot occur. Findings from serial aBL exposure to infected mouse wounds suggest that patients that are treated for dermatological infections may potentially withstand sequential aBL treatments without resistance development becoming an issue. However, as mice were only subjected to 5 cycles of repeated aBL exposure, we may only conclude that 5 cycles of sub-curative aBL exposure does not induce resistance development.

## Author Contributions

OH, YF, IA, and XG performed the *in vitro* serial exposure studies. LL performed the *in vivo* studies. LL and TD designed the study, carried out data analysis and wrote the paper.

## Conflict of Interest Statement

The authors declare that the research was conducted in the absence of any commercial or financial relationships that could be construed as a potential conflict of interest. The reviewer AP and handling Editor declared their shared affiliation at time of review.
